# Retracted: Optimization, Composition, and Antioxidant Activities of Exo- and Intracellular Polysaccharides in Submerged Culture of *Cordyceps gracilis* (Grev.) Durieu & Mont.

**DOI:** 10.1155/2018/2980342

**Published:** 2018-05-02

**Authors:** Evidence-Based Complementary and Alternative Medicine

Evidence-Based Complementary and Alternative Medicine has retracted the article titled “Optimization, Composition, and Antioxidant Activities of Exo- and Intracellular Polysaccharides in Submerged Culture of* Cordyceps gracilis* (Grev.) Durieu & Mont.” [1] because of concerns regarding data integrity and close similarities between this article on polysaccharide yields from* Cordyceps gracilis* cultures and two other articles [2, 3] by the authors on other members of this fungal genus,* Cordyceps cicadae* and* Cordyceps tuberculata*:

- Sharma S. K., Gautam N., and Atri N. S., “Optimized extraction, composition, an antioxidant and antimicrobial activities of exo and intracellular polysaccharides from submerged culture of Cordyceps cicadae,” BMC Complement. Altern. Med. 15: 446 (2015).
- Sapan Kumar Sharma and Nandini Gautam, “Bioprospection of cordyceps tuberculata for production of bioactive polysaccharides under submerged culture conditions,” EPRA International Journal of Research and Development (IJRD), 1:10, December 2016.

The methods for the Evidence-Based Complementary and Alternative Medicine and BMC Complement. Altern. Med articles are highly similar, except for the beginning. The results and discussion sections in these articles are similar and many of the same phrases were used. Because of this we asked the authors for the raw data, which the corresponding author Dr. Sharma provided. We also asked the Department of Botany at Punjabi University to provide photographs and descriptions of two herbarium specimens, PUN 6964 and PUN 7194, which they said were submitted to their herbarium by Sapan Kumar Sharma on 28.08.2014 and 10.08.2015, respectively. They provided scanned images of herbarium vouchers and specimens.

The concerns are as follows:

1. Figure 1(b) in each article presents the same photograph, though they are meant to represent different fungal species.
2. Tables 1 and 2 in each article show many exact digit matches between the articles for the polysaccharide yield means and standard errors, especially in Table 1, despite the results representing different species.
3. The distribution of insignificant digits (i.e., digits unimportant to the measured value) is expected to be uniform [4]. The decimal values in the polysaccharide yield means and standard errors in Tables 1 and 2 in the three articles have a significantly nonuniform distribution.
4. The scanned laboratory notebooks have no dates or signatures. The raw data for the polysaccharide yield replicates do match the published means and standard errors, but this means there is a perfect match between the means and standard errors for the IPS yields by temperature between the articles in Evidence-Based Complementary and Alternative Medicine and BMC Complement. Altern. Med, even though these results correspond to different values in the underlying data. The decimal values in the raw data for the IPS yields by temperature also have a significantly nonuniform distribution.
5. One of the Evidence-Based Complementary and Alternative Medicine article authors, Dr. Narender Singh Atri, said the email address used for him for the submission is not his, but he is an author of these articles in Evidence-Based Complementary and Alternative Medicine and BMC Complement. Altern. Med.

The corresponding author, Dr. Sharma, does not agree with retraction.

## References

[1] Sharma S. K., Gautam N., Atri N. S. (2015). Optimization, composition, and antioxidant activities of exo- and intracellular polysaccharides in submerged culture of *Cordyceps gracilis* (Grev.) durieu & Mont..

[2] Sharma S. K., Gautam N., Atri N. S. (2015). Optimized extraction, composition, antioxidant and antimicrobial activities of exo and intracellular polysaccharides from submerged culture of Cordyceps cicadae.

[3] Sharma S. K., Gautam N. (2016). Bioprospection of cordyceps tuberculata for production of bioactive polysaccharides under submerged culture conditions.

[4] The Office of Research Integrity https://ori.hhs.gov/statistical-forensics-check.

